# Locomotion of the C_60_-based nanomachines on graphene surfaces

**DOI:** 10.1038/s41598-021-82280-7

**Published:** 2021-01-28

**Authors:** Seyedeh Mahsa Mofidi, Hossein Nejat Pishkenari, Mohammad Reza Ejtehadi, Alexey V. Akimov

**Affiliations:** 1 Institute for Nanoscience and Nanotechnology (INST), Sharif University of Technology, 14588-89694 Tehran, Iran; 2 Mechanical Engineering Department, Sharif University of Technology, 11155-9567 Tehran, Iran; 3 Department of Physics, Sharif University of Technology, 11155-9161 Tehran, Iran; 4 Department of Chemistry, University at Buffalo, State University of New York, Buffalo, 14260-3000 USA

**Keywords:** Physical chemistry, Surface chemistry, Nanoscale materials, Theory and computation, Atomistic models, Graphene, Molecular machines and motors, Two-dimensional materials

## Abstract

We provide a comprehensive computational characterization of surface motion of two types of nanomachines with four C_60_ “wheels”: a flexible chassis Nanocar and a rigid chassis Nanotruck. We study the nanocars’ lateral and rotational diffusion as well as the wheels’ rolling motion on two kinds of graphene substrates—flexible single-layer graphene which may form surface ripples and an ideally flat graphene monolayer. We find that the graphene surface ripples facilitate the translational diffusion of Nanocar and Nanotruck, but have little effect on their surface rotation or the rolling of their wheels. The latter two types of motion are strongly affected by the structure of the nanomachines instead. Surface diffusion of both nanomachines occurs preferentially via a sliding mechanism whereas the rolling of the “wheels” contributes little. The axial rotation of all “wheels” is uncorrelated.

## Introduction

Natural molecular machines are in the heart of the cellular machinery of living organisms, performing complex vital functions, and transferring materials with high efficiency^1–3^. These biomolecular systems inspired the development of artificial machines that function at the molecular level^4,5^. The widespread function of the controlled molecular motion in fundamental natural processes suggests that notable rewards can be gained by improvements of synthetic molecular machines^6,7^. Nanocars constitute one example of artificial molecular machines with chassis, axles, and wheels designed for nanoscale transport on various surfaces^8,9^. Understanding the molecular motion on surfaces is essential for controlling the dynamics and functioning of molecular machines^10^. In particular, one of the long-standing questions to address is the relationship between the design of nanomachines and their diffusion properties^11,12^.

Synthetic chemists suggested a variety of structural designs intended to increase the mobility of nanocars on surfaces^13,14^. Tour and colleagues synthesized nanocars specifically for the transportation of other molecules. They built a variety of nanocars that consisted of chassis and wheels. The spherical shape and stability of Buckminsterfullerene, C_60_, motivated them to use fullerene moieties as the first types of wheels for such Nanocars and Nanotrucks^15–18^. A number of computational studies of nanocars’ motion on a variety of substrates were reported in the past, including C_60_-based Nanocars and Nanotrucks on metal surfaces using either the rigid-body^19–22^ or all-atomic^23,24^ molecular dynamics (MD) methods. Nemati and co-workers investigated the role of vacancies^25^, impurities^26^, and step-like surface defects^27^ to control the diffusion of C_60_ and C_60_-based nanocars. Lavasani’s group^28,29^ demonstrated how the chassis structure was affecting the diffusive motion of carborane-wheeled nanocars on a gold surface.

In the past decade, all-carbon-based materials such as few-layers graphene, graphyne, carbon nanotubes, or graphene nanoribbons have been recognized for their unique electronic and structural properties^30^, making them promising materials for a wide range of applications. In particular, graphene can be considered a potential surface for nanocar operation in nanoscale molecular transporting applications. Such potential applications recently stimulated studies of nanocars on such all-carbon based material surfaces. Ejtehadi and co-workers^31,32^ studied the diffusive motion of C_60_ on graphene and on a variety of graphyne structures^33^. Savin et al.^34^ characterized the thermally-induced diffusion of C_60_ fullerene on graphene nanoribbons. Jafary-Zadeh et al.^35^ created a transporting pathway on graphene to confine the diffusive motion of C_60_. Ganji et al.^36^ theoretically investigated the motion of a carborane-wheeled nanocar on graphene and graphyne surfaces using the density functional theory.

Monolayers and few-layers of graphene are not flat and exhibit a wavy morphology of the surface, with ripples and out-of-plane deformations^37^. As observed by the scanning tunneling microscopy, the thermally-induced ripples in graphene form standing waves that evolve erratically^38^ Such ripple waves could in principle affect the motion of nanocars due to occasional variation of the contact level and interlock effects to hinder molecule translation^39^. Although several works reported studies of the motion of a single molecule (e.g. C_60_) on graphene^32,34,40–42^, the dynamics of nanocars on the flexible graphene surface that can form ripples has not been investigated yet. Furthermore, little work has been done to delineate the role of the chassis rigidity in the surface dynamics of nanomachines. To the best of our knowledge, no such studies have been reported for nanomachines moving of graphene surfaces.

In this work, we study several effects that can control the dynamics of nanomachines on graphene surfaces: (1) the role of substrate flexibility; (2) the role of the chassis flexibility. We do this by comparing the results of all-atomic molecular dynamics in a variety of atomistic models. To examine the first effect, we consider the motion of nanocars on single-layer graphene (SLG) and frozen layer graphene (FLG) surfaces. To examine the second effect, we choose two nanocars with four C_60_ wheels in each, but different in their chassis rigidity: the Nanocar and Nanotruck.

## Computational methodology

**Figure 1:**
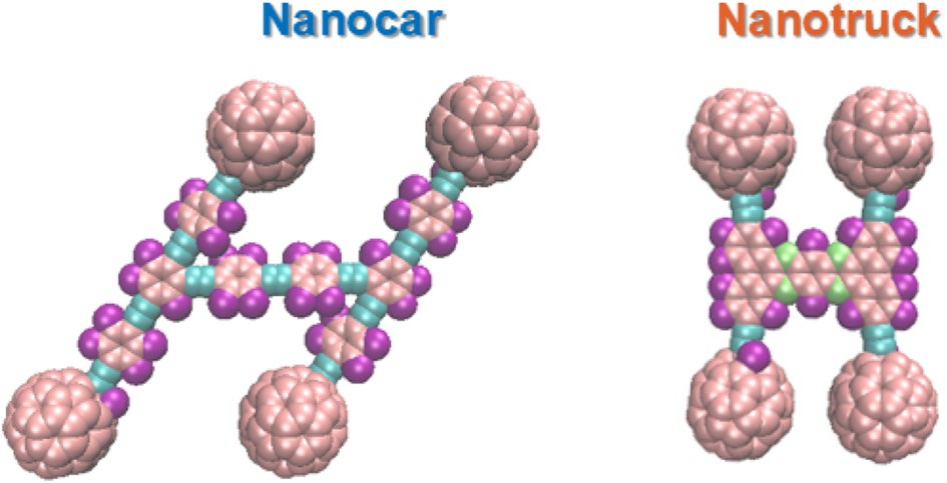
Atomic structure of Nanocar with Flexible chassis, size of 3 × 4 nm^2^, and 3 atom types (left side); and Nanotruck with Rigid chassis, size of 2 × 3 nm^2^, and 4 atom types (right side). Color codes: tan—sp2 and sp3 carbon, (C); teal—sp carbon (C); purple—hydrogen (H); green—nitrogen (N). The structures are visualized by VMD^44^.

**Figure 2:**
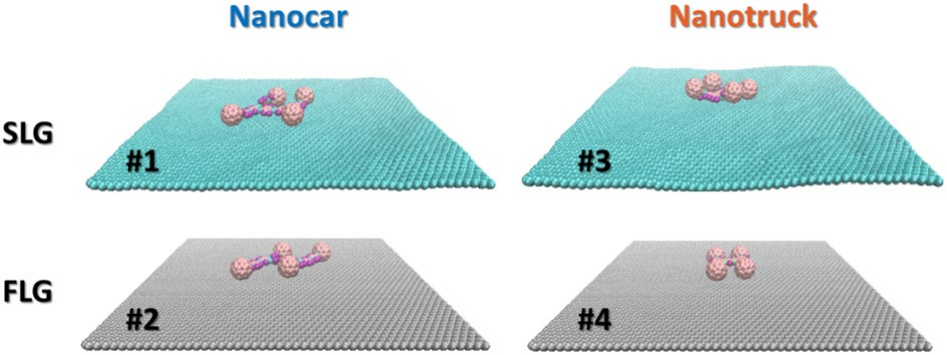
Representation of the four types of molecule/substrate systems used in the present work. Fixed atoms are demonstrated in gray color. System type 1 (NC/SLG), Nanocar (NC) on the single-layer graphene substrate where long-range ripples of graphene can be observed. System type 2 (NC/FLG), all substrate atoms are kept fixed. System type 3 (NT/SLG), Nanotruck (NT) on the single-layer graphene substrate where long-range ripples of graphene can be observed. System type 4 (NT/FLG), all substrate atoms are kept fixed. The structures are visualized by VMD^44^.

We study the motion of two types of previously synthesized fullerene-based machines^18,43^ with C_60_ wheels (Fig. 1): (a) a flexible Nanocar (NC, C_310_H_34_), a 3 × 4 nm molecule; and (b) a rigid chassis Nanotruck (NT, C_282_H_18_N_4_) a 2 × 3 nm one. To be able to study graphene flexibility (surface ripples) and elucidate the effects, we define two types of substrates:

- Single-layer of graphene (SLG) in which all of graphene’s atoms are allowed to move, which leads to the surface ripple formation. This substrate is the best approximation of the SLG that can be fabricated experimentally. The absence of vertical interactions with other layers (as would be the case for graphite or multi-layer graphene), leads to the formation of pronounced ripples (Fig. 2, top panels).
- Single-layer of graphene with the frozen motion of all atoms (frozen layer graphene, FLG). This design corresponds to a hypothetic case of an ideally flat graphene surface, without ripples and without thermal motions.

By considering the motion of Nanocar and Nanotruck on two graphene substrate types, we have four systems to study (Fig. 2). To study the motion of the fullerene-based nanomachines on graphene substrates, we utilize the all-atom classical molecular dynamics (MD) simulations. The substrates are modeled as 12 × 12 nm^2^ square sheets containing 5744 carbon atoms. The graphene sheet is positioned at $z=0$ plane. Periodic boundary conditions are applied in x and y directions to allow unlimited diffusion of the molecules over the surfaces.

For visualizing the initial and output structures we have used VMD 1.9.2 software package^44^ (http://www.ks.uiuc.edu/Research/vmd/). Molecular interactions are described using classical force fields as implemented in the Largescale Atomic/Molecular Massively Parallel Simulator (LAMMPS 22Aug2018 version) software^45^. Tersoff potential is used to describe covalent bonds in graphene and nanomachines. Lennard–Jones 6–12 (LJ6-12) potential is used to describe the non-bonded interaction between each atom of graphene with each atom of nano-machine:

1 $${U}_{ij}=4{\varepsilon }_{ij} \left[{\left(\frac{{\sigma }_{ij}}{{r}_{ij}}\right)}^{12}-{\left(\frac{{\sigma }_{ij}}{{r}_{ij}}\right)}^{6}\right] {r}_{ij}<{r}_{cut},$$

Here, σ and ε are the van-der-Waals (vdW) radius and depth of atomic interaction potential for each pair of species, respectively. The parameters related to each atom pairs are listed in Table 1 based on previous studies on non-bonding interactions^46–49^. The cut-off radius, ${r}_{cut}$ of 12 Å is utilized in this work to reduce computational expenses. This selection is motivated by the commonly used criterion, ${r}_{cut}>2.5\sigma$.

**Table 1:** Lennard–Jones interaction parameters^46–49^.

Atom pair	Ε (meV)	σ (Å)
C–C	2.41	3.4
H–H	1.449	2.65
N–N	2.597	3.416
C–H	1.337	2.81
N–C	2.501	3.408
N–H	1.973	3.033

MD trajectories are integrated for 40 ns for each system in the NVT ensemble. The velocity Verlet integration scheme with the integration time step of 1 fs is used. Data recorded every 200 timesteps, resulting in 200,000 data points for each simulation. The Nose–Hoover thermostat is used to maintain the target bath temperature. The thermostat damping parameter that determines the rate of heat exchange between the system and the thermostat (Tdamp) is set to 100 fs, which is a typical value used in molecular simulations^50^. For each system, the simulations are performed for temperatures ranging from 5 to 1000 K.

To compute the desorption temperatures, we utilize the following procedure. At high temperatures, the molecule can escape the substrate by overcoming the van-der-Waals energy and move away to distances greater than 30 Å where the interaction energy becomes negligible. Therefore, we treat the molecules that move away from the surface by 30 Å or more and never return during the simulation (40 ns) as a desorbed case. When we observe such “desorption” events, we reduce the temperatures by 25 K to examine nearby temperature values and test whether desorption still occurs at lower temperatures. If the desorption process is still observed, we lower our estimate of the desorption energy and repeat the process. Otherwise, we consider the obtained value as the resulting desorption temperature with a 25 K error bar.

To quantify the translational motion of nanomachines, we compute the mean square displacement (MSD) for every type of simulation (as defined by the system type and MD conditions). MSD quantifies the mobility of molecules due to random-walk-like motion. The MSD is used to compute the surface (2D) diffusion coefficient, $D$, via^51^:

2 $$MSD\left(t\right)=\langle {\left(x\left(t\right)-x\left(0\right)\right)}^{2}+{\left(y\left(t\right)-y\left(0\right)\right)}^{2}\rangle =4D{t}^{\alpha }$$

Here, $x$ and $y$ are coordinates of the molecule center of mass, $D$ and $\alpha$ are the 2D diffusion coefficient and the diffusion anomaly parameter, respectively. For normal diffusion, the latter parameter is close to unity, $\alpha =1$, whereas for super- and sub-diffusion regimes $\alpha >1$, and $\alpha <1$, respectively. The angle brackets indicate the ensemble averaging, which is performed in the following manner. The initially obtained 40 ns trajectory consists of 200,000 data points (${N}_{T}$). It is split into 60 intervals (${N}_{seg}$), each containing 660 ps and $\frac{{N}_{T}}{{N}_{seg}}\approx 3333$ data points each. These intervals are regarded as 60 independent (sub)-trajectories, each started with a distinct initial condition, sampled from the NVT ensemble (by the NVT MD of the original long trajectory). Finally, the averaging over the 60 sub-trajectories is used to compute the MSD in Eq. 2. Each MSD is computed as the function of time up to 660 ps. The approach follows closely the recipe of Ernst and Kohler^52^.

## Results and discussion

### Vertical motion

**Figure 3:**
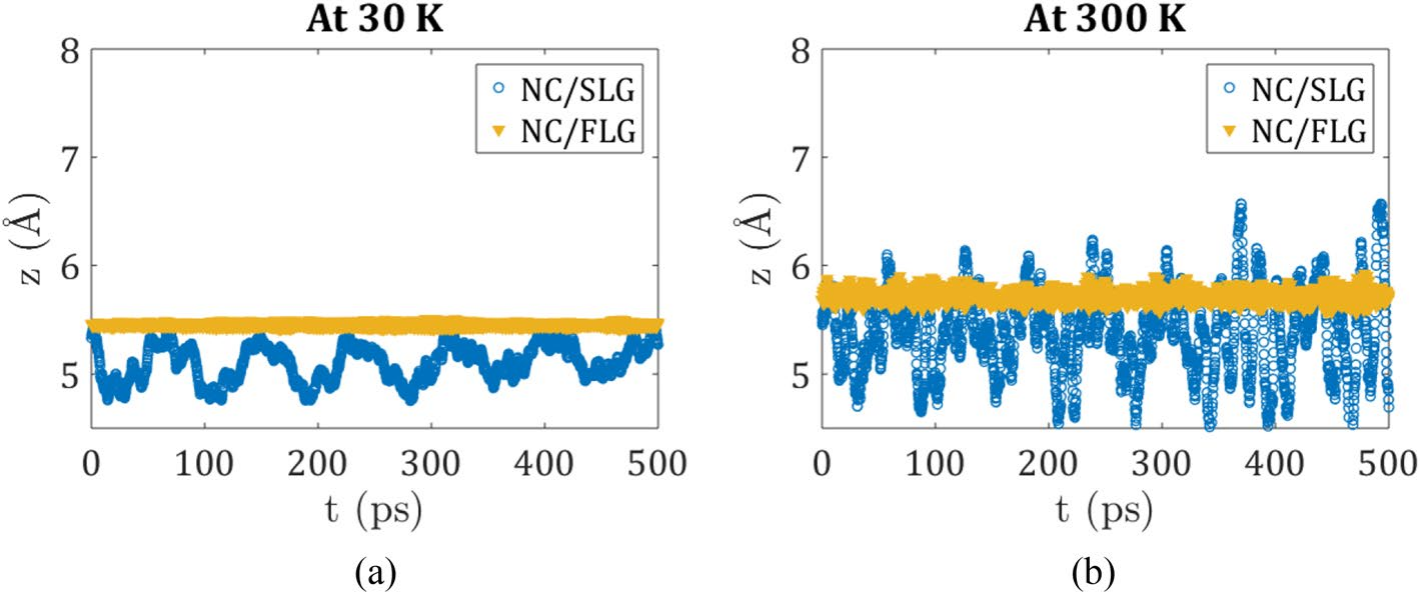
Variation of z coordinate of Nanocar center of mass during 500 ps for two types of substrate at (**a**) 30 K; (**b**) 300 K.

The displacement of the molecule in the vertical direction may facilitate lateral diffusion since the lateral displacement barriers would decrease when the molecule moves away from the surface. This type of motion is temperature-activated. Figure 3 demonstrates the variation of z-component of Nanocar COM along representative trajectories, which corresponds to the vertical height of Nanocar on top of the substrates at two different temperatures. The same trend is seen for the vertical motion of a Nanotruck on graphene substrates.

At low temperatures (e.g. 30 K, Fig. 3a), the Nanocar COM is mainly located 5.5 Å away from the surface. Due to the larger number of atoms and larger interaction energy in nanomachines as opposed to C_60_, the average distance is smaller for the former than for the latter, reported earlier to be 6.4 Å^32,53^. Already at this temperature, one may observe the signs of surface rippling as manifested in larger molecule height fluctuation for the flexible SLG as opposed to the FLG system with constraints on the motion of surface atoms. Surface ripples intensify the oscillation of the molecule around the equilibrium distance. Even at a low temperature, the height of the molecule shows the trace of the graphene surface ripples. The surface rippling effect is enhanced with temperature, leading to the z value of Nanocar COM to fluctuate stochastically between 4.5 and 6.5 Å (Fig. 3b). This long-range fluctuation may put the molecule in a highly repulsive region of interaction energies. However, the z coordinate of the surface itself changes due to rippling, so when the surface ripples down (in the negative z-direction), the molecule can dip together with it, leading to z coordinates of COM down to 4.5 Å. Under these conditions, the nanomachine/graphene equilibrium distance is still notably larger than 4.5 Å.

The Nanocar COM z coordinate oscillations lead to more probable desorption of the molecule from the substrate. We examine the affinity for Nanocar and Nanotruck to desorb from the substrate based on the outcomes of the 40 ns MD simulations for each type of system in a range of temperatures higher than 1000 K (no desorption occurred at temperatures under 1000 K). For each combination of temperature and system, the simulations are repeated 3 times. Each repetition corresponds to a distinct initial velocity distribution (different seed numbers). We find that the desorption temperatures for both systems are comparable (Table 2). For both systems, the graphene surface rippling decreases the desorption temperature. For both systems, the desorption temperatures are notably higher than for a single C_60_ molecule, which can be attributed to the bare number of atoms being larger in both NC and NT than in fullerene. The increased substrate/adsorbate interaction strength for the former also correlates with the smaller z distances of the COM for these systems as opposed to the fullerene. Ulbricht et al.^54^ experimentally showed that the C_60_ desorbed from few-layered graphite at 580 K, which is in good correspondence with the 550 K temperature obtained by SLG dynamics and interactions. The desorption temperature for C_60_ from the flat FLG (850 K) is notably larger than the experimental value. This remarks that the inclusion of the rippling effects is crucial for computing an accurate description of thermodynamics of the interaction of molecules with graphene.

**Table 2:** Estimated desorption temperatures (K) in 4 studied systems and an isolated C_60_ wheel.

Molecule/substrate type desorption temperature (K)	SLG	FLG
Nanotruck	1575 ± 25	1700 ± 25
Nanocar	1500 ± 25	1625 ± 25
C_60_^53^	550 ± 25	850 ± 25

Our interpretation of the observed trends is as follows. On the more flexible SLG surface, the average curvature at the point of surface/adsorbate contact is non-zero. As a consequence, some parts of the nanocar molecule (those away from the contact, where the surface is bent away from the molecule) are positioned farther than the optimal distance, whereas other parts of the molecule (at the contact, zero curvature) would prevent the molecule from getting even closer to the surface. This picture changes dynamically—the “close contact” regions become the “bent-away” ones, changing back and forth over time. As a result, the average substrate/adsorbate energy decreases in comparison to what it could be on the ideally flat surface, leading to decreased desorption energies. On the contrary, on the ideally flat surface (as modeled here by the FLG system), the interactions are maximized due to the absence of the “bent-away” regions.

Our calculation on graphene ripples during the time evolution of SLG simulations shows that ripples amplitude (standard deviation of z components of graphene atoms) is increasing with temperature and saturate to about 1 Å at high temperatures. The average peak-to-peak distance of the ripples is computed to be 25 ± 5 Å, which is on the order of the Nanocar and Nanotruck size. The ratio of the ripple peak-to-peak distance to the nanomachine’s size is also consistent with our rationalization of the desorption temperatures.

The peak-to-peak distances computed in this work are in good agreement with previous theoretical and experimental studies on rippled graphene, which reported the graphene roughness to be between 75.2 to 109.0 pm^55^. Thomsen et al.^56^ directly measured the freestanding graphene roughness (ripple amplitude) as about 1.14 Å using diffraction tilt analysis in the transmission electron microscope (TEM) method. Kirilenko et al.^57^ reported that a graphene roughness root mean square of $\sqrt{<{h}^{2}>}=1.7 \AA$. Li et al.^58^ reported the peak heights of the rippled graphene to range from 0.2 to 0.4 nm and periodicities (peak-to-peak distance) to range from 3 to 10 nm.

### Lateral diffusion

**Figure 4:**
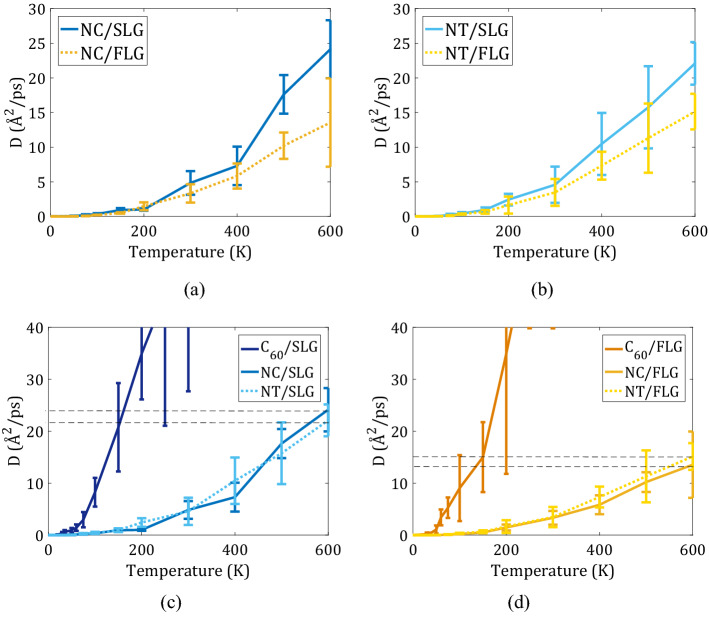
Diffusion coefficient as a function of the temperature diagram of the (**a**) Nanocar and (**b**) Nanotruck mobility on two substrates: (SLG—blue, FLG—yellow). Diffusion coefficient of Nanotruck, Nanocar, and a C_60_ wheel on (**c**) SLG, and (**d**) on FLG.

To quantify the lateral diffusion of Nanocar and Nanotruck on graphene with and without surface ripples, we compute the diffusion coefficients for all systems for a range of temperatures (Fig. 4). The raw data for such calculations are summarized in the Supplementary Information (Figure S1 for the representative MD trajectories; Figure S2 for the MSD vs. time). Our analysis suggests that the anomaly coefficient depends neither on the surface flexibility nor on the nanomachine’s structure and is primarily a function of temperature (see Supplementary Fig. S3). Unlike the anomaly coefficient, the diffusion coefficients depend notably on the flexibility of the substrate, (Fig. 4, panels a and b) and, to a smaller extent, on the nanomachine’s structure (Fig. 4, panels c and d). In particular, we find that the diffusion coefficients increase for both adsorbates on the more flexible SLG surface as opposed to the planar FLG.

The flexible freestanding graphene (SLG) surface show bending opposite to the fixed graphene (FLG). This bending phenomenon is observable in experimental microscopy^59^. In the view of static interactions, like a “lock-key” or “host–guest” type, the relationship between the molecule mobility and the flexibility of surface is counterintuitive; “more bent” supposedly means stronger molecule/SLG interactions or lower energy level. However, since the ripples on 2D graphene are dynamic (proved by experiment and STM imaging^38^), the surface bends dynamically so that the energetically favorable points are transient and quickly turn into energetically unfavorable configurations. The fluctuation of potential energy profile (shifting of minimum position) in time due to rippling (opposite of the constant profile of the ideally flat graphene), creates configurations that have negative potential energy value at one time suddenly turn to positive potential value at another time. Hence, the potential energy is averagely more positive in the system with a flexible substrate. Consequently, the desorption energy decreases, resulting in higher mobility and diffusion coefficient of the molecule.

The computed diffusion coefficient for Nanocar and Nanotruck on SLG is on the order of 5 Å^2^/ps at room temperature. This value is an order of magnitude less than the room-temperature diffusion coefficient (50 Å^2^/ps) for C_60_ on a graphene sheet^53^. We attribute this difference to the greatly increased adsorbate/surface interaction energy for the NC or NT moving on graphene monolayer, as compared to a single C_60_ molecule moving of such surface. However, the molecular size is not the only determinant of the molecule’s diffusion coefficient. If an increase of the molecule’s size is associated with its vertical (normal to the surface) elongation, the atoms that are more distant from the surface would contribute only little to the surface/adsorbate interaction energy due to the short-range nature of the van der Waals interactions. Thus, having the planar alignment of most atoms in the nanomachines is an effective way to slow down the machine’s diffusion^60^.

**Figure 5:**
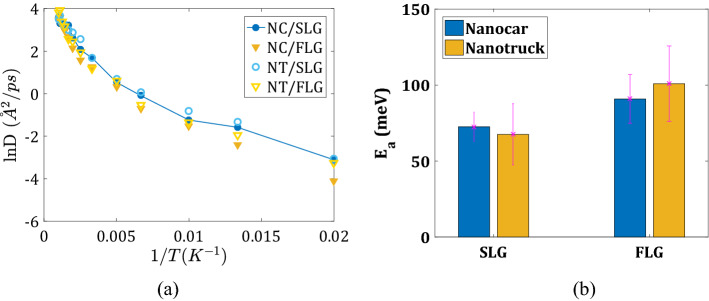
Quantification of nanomachines’ lateral mobility: (**a**) Arrhenius plot of Nanocar and Nanotruck diffusion coefficients on two substrate types (SLG—blue, FLG—yellow); (**b**) activation energies computed for all systems.

Using the Arrhenius plot and diffusion coefficients at high temperatures (Fig. 5a, also see Supplementary Fig. S4a), we compute the activation energies for the diffusion of both nanomachines on all surfaces (Fig. 5b). The activation energy of 2D diffusion is 72.58 meV for Nanocar on SLG and 90.88 meV on FLG, 67.56 meV on SLG, and 100.90 meV on FLG (Fig. 5b). The activation energy for the NC and NT molecules are comparable to each other for a given type of substrate. They are generally lower on the flexible surface, SLG, compared to the ideally planar FLG. These energies are 2–2.5 times larger than the activation energy for the diffusion of the C_60_ molecule (39.2 meV), suggesting that the fullerene wheels as a part of nanocars do not interact as strongly with the substrate as they would be on their own. In other words, combining them via the chassis facilitates the motion of 4 fullerene wheels together. The larger activation energies on the FLG than they are on the SLG are consistent with the above “bending-away” explanation of the differences in the activation energies for desorption.

### Rotational motion

The rotational motion of nanomachines on the surfaces is characterized by the temporal evolution of the components of angular velocity ($\omega$) as the function of time. The cumulative rotational angles are calculated as $\theta \left(t\right)={\int }_{0}^{t}\omega \left(t{^{\prime}}\right)dt{^{\prime}}$ to the rotational diffusion (see Supplementary Fig. S5). We focus on the components that correspond to horizontal (cartwheeling, ${\omega }_{x}$) and vertical (pivoting, ${\omega }_{z}$) rotation of the molecule around its center of mass. No significant horizontal rotation occurs, due to the structure of the nanocars. Only short-lived fluctuations of the cartwheeling rotation angle as observed, which is attributed to the dynamical change of the instantaneous structure of the nanocars.

**Figure 6:**
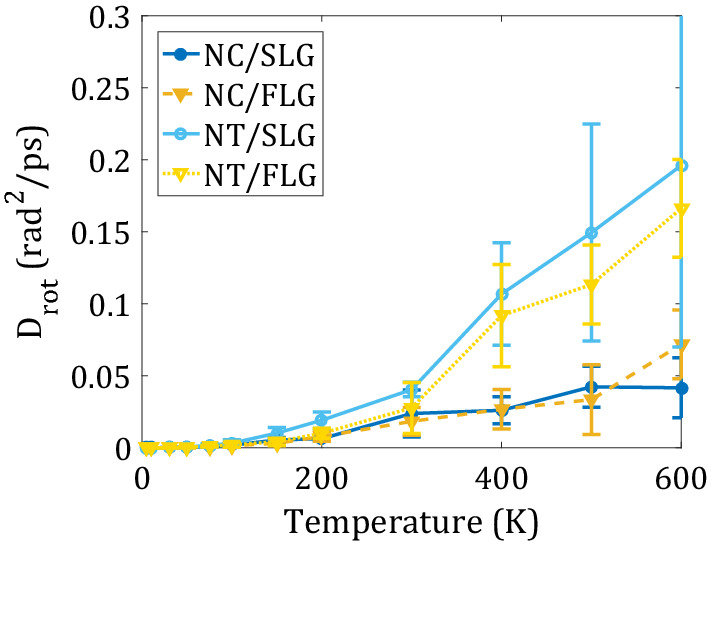
Rotational diffusion coefficient around the vertical axis as a function of temperature. Nanotruck rotates faster than flexible Nanocar.

Expectedly, the major type of rotation of both molecules exhibit is the pivoting motion, which changes the car’s yaw angle. We quantify the pivoting diffusion by computing the corresponding diffusion coefficients at the range of temperatures for all systems (Fig. 6). Unlike the translational diffusion, the dependence of the pivoting diffusion on the type of substrate is much weaker. Instead, the structure of the adsorbate is the dominant factor. Interestingly, the NT has higher pivoting diffusion coefficients than NC. This observation can be attributed to a more rigid structure of the NT molecule, making the collective pivoting motion easier to coordinate. For the more flexible NC molecule, there is a higher degree of independence for all parts, making their collective pivoting a more difficult task.

**Figure 7:**
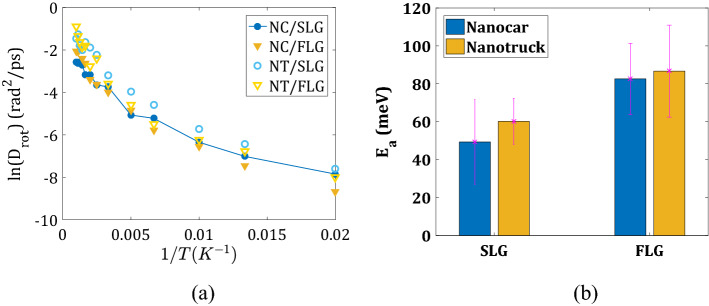
Quantification of rotation about normal z-axis: (**a**) Arrhenius plot for Nanocar and Nanotruck diffusion coefficients on two substrate types (SLG—blue, FLG—yellow); (**b**) activation energies computed for all systems.

The activation energies for pivoting motion are computed using the Arrhenius plot of the corresponding diffusion coefficients (Fig. 7, panels a and b). We find these values to be of the same order of magnitude as the activation energies for the translational motion of the nanomachines. Analogous to the activation energies for translation, they are smaller for the flexible graphene surface (SLG system): 49.33 meV for Nanocar and 60.19 meV for Nanotruck. On the planar FLG surface, they are increased: 82.60 meV for Nanocar and 86.67 meV for Nanotruck. The increase of the activation energies on the FLG as opposed to SLG can be understood in terms of increased/strengthened surface/adsorbate interactions and smaller flexibility for the constrained system to be able to find smaller-energy pathways.

### Wheels rolling

**Figure 8:**
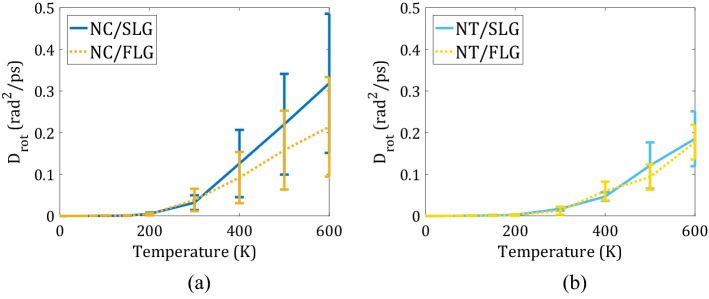
Rotational diffusion coefficient of wheels in (a) Nanocar and (b) Nanotruck as a function of temperature. Wheels in flexible Nanocar rotate more easily than in Nanotruck.

We calculate the average rotational MSD of all four wheels in each nanomachine (Fig. 8, panels a and b). Within the timescales of simulation, we do not observe any wheel rolling for temperatures below 200 K, neither for Nanocar nor for Nanotruck. The diffusion coefficient of the rotation of wheels in Nanocar is 1.5 times larger than that for wheels in Nanotruck. For the Nanotruck, the presence of surface ripples doesn’t affect the rotational diffusion coefficient, whereas for the Nanocar the ripples facilitate the rolling of the wheels.

**Figure 9:**
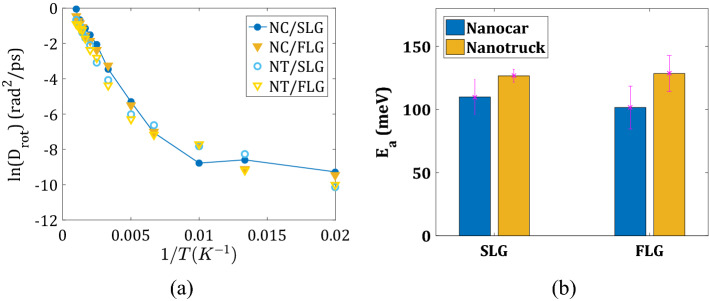
Quantification of the rotation of wheels: (**a**) Arrhenius plot of diffusion coefficient for wheels rolling in Nanocar and Nanotruck on two substrate types (SLG—blue, FLG—yellow); (**b**) activation energies computed for all systems.

Using the Arrhenius plot of the wheels’ rolling diffusion coefficient (Fig. 9a), we estimate the activation energy for wheel rotations (Fig. 9b). The activation energies are determined mainly by the nature of the chassis and not by the type of substrate. For Nanocar, it is 110.00 meV on SLG and 101.63 meV on FLG. For Nanotruck, it is 126.71 meV on SLG and 128.59 meV on FLG. Such results are easy to understand—the rolling of the wheels is determined by all the local steric hindrance around them, which primarily stems from the structure of the nearby chassis. In the NT, the wheels are located closer to the chassis and are more hindered. The chassis of the NC, on the other hand, is quite flexible which minimizes the steric hindrance of the wheels when they roll. As a consequence, the activation energies for rolling in the NC system are lower as compared to those in the NT.

There are two major mechanisms of the nanomachine’s motion: sliding and rolling. To quantify the contribution of each mechanism to the motion of Nanocar and Nanotruck, we compute the slip ratio^61,62^:

3 $$Slip ratio=\left| \frac{{\omega }_{w}\times {r}_{w}- \stackrel{-}{{V}_{w}}}{\stackrel{-}{{V}_{w}}}\right|$$

**Figure 10:**
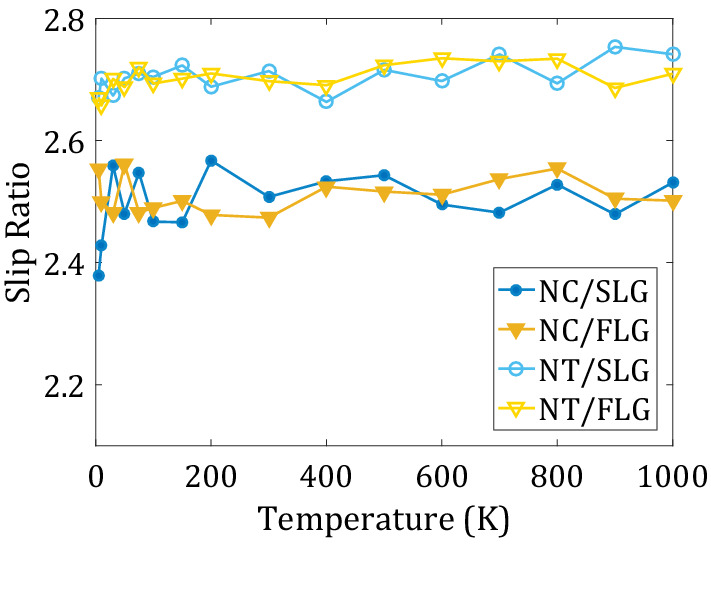
Average of wheels slip ratio in nanomachine/surface systems at different temperatures.

Here, $\stackrel{-}{{V}_{w}}$ is the translational velocity vector of COM of the wheel. The slip ratio is a measure of sliding vs. rolling which is commonly used in the automobile industry to calculate drift in a tire motion of macroscopic machines. If this ratio approaches zero, the wheels undergo a pure rolling type of motion and no drift (or sliding) occurs. The slip ratios computed for each of the four systems fall in the 2.4–2.8 range for all temperatures (Fig. 10). This means the sliding motion is the dominant diffusion mechanism. Interestingly, the ratio is slightly larger for the NT than for the NC, indicating that the former may involve smaller wheel rolling motion. This observation is consistent with larger activation energies for the wheel rolling in the NT system. The slip ratios are practically independent of the surface flexibility/roughness or temperature.

As discussed in our previous study^53^ the energy variation of sliding motion of a C_60_ molecule on graphene is about 1.4 meV while the rolling of C_60_ around the horizontal axis was as large as 40 meV. Comparing these energies clarifies that a C_60_ tends to slide than to roll on graphene. Here, the same result was obtained for both types of four C_60_-wheeled nanomachines showing the major role of wheels-surface interactions in the motion mechanism.

To investigate whether the rotation of the wheels in the nanomachines considered is correlated, we compute the wheels correlation function^28^:

4 $$C=\frac{2}{3 N(N-1)} \sum_{i=1}^{N}\sum_{j>i}^{N}\sum_{k=x, y, z}|\rho ({\omega }_{ k,i}, {\omega }_{ k,j})|$$

**Figure 11:**
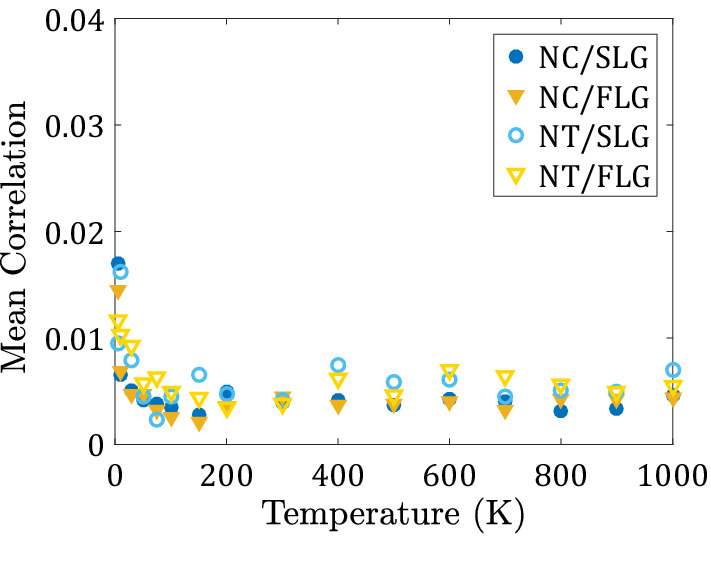
Mean correlation of the rotation of wheels in nanomachine/surface systems at different temperatures.

Here, $N$ is the number of wheels, ${\omega }_{k,i}$ is the $k$ th ($k=x,y,z$) component of the angular velocity vector of the wheel $i$, and $\rho \left({\omega }_{ k,i}, {\omega }_{ k,j}\right)$ is the pairwise linear correlation coefficient^63^ between each pair of wheels. The correlation function, Eq. (4) is defined in such a way that if its value approaches 1, there is a perfect correlation of rolling of all the wheels with each other, and if its approaches 0, there is no correlation among any of the wheels. Our calculation indicates that the correlation function Eq. (4) is negligibly small for all systems at all temperatures (Fig. 11). Thus, the wheels in Nanocar and Nanotruck roll completely independently of each other. This result is consistent with the analogous calculations reported for nanocars moving on gold surfaces^28^.

## Conclusions

We demonstrate that both the surface and chassis flexibility critically affect the dynamics of nanomachines on graphite surfaces. The presence of surface ripples increases the magnitude of the nanocar/substrate vertical distance fluctuations and decreases the desorption temperature, regardless of the substrate molecule (Nanotruck, Nanocar, and C_60_). As a consequence, the surface diffusion coefficients are amplified on the flexible graphene surface for both nanomachines. The corresponding activation energies decrease by 20% (from 90.88 to 72.58 meV) for Nanocar and by 33% (from 100.90 to 67.56 meV) for Nanotruck on the flexible graphene as compared to the frozen surface.

We find that on the graphene surface, the Nanotruck has a higher rotational diffusion coefficient than the Nanocar. Nanotruck can rotate even faster on SLG compared to FLG while surface type does not notably change Nanocar’s rotation. The rotational activation energies depend primarily on the surface type. Similar to activation energies for translational diffusion, these energies are lower for the flexible surfaces. For Nanocar, these energies are 49.33 meV on SLG and 82.60 meV on FLG. For Nanotruck, they are 60.19 meV on SLG and 86.67 meV on FLG.

We quantify the axial rotational diffusion of wheels in the two types of nanomachines. We find that the corresponding diffusion coefficients are 1.5 times larger in the Nanocar compared to that in the Nanotruck. Such differences correlate with the difference in the activation energies for the axial rotation of the wheels. We observe no significant influence of surface flexibility on the activation energies of the wheels’ rotation. On the contrary, the molecule’s structure becomes plays the dominant role here. The computed values are smaller for more flexible Nanocar molecule (110.00 meV on SLG and 101.63 meV on FLG) than for more rigid Nanotruck structure (126.71 meV on SLG and 128.59 meV on FLG). The sliding mechanism is found to be dominant over the rolling one for both types of nanomachines, although more pronounced in the Nanotruck, where the wheels’ rotation is hindered to a larger extent. We demonstrate that the axial wheels’ rotation in Nanocar and Nanotruck is uncorrelated—each wheel rotates completely independently of all other wheels.

## Supplementary Information

Supplementary Information.

## Data Availability

The computational protocols used in this work, the key input and output files, as well as important structural data are available online at https://github.com/AkimovLab/Project_Nanocar. The repository also provides the digital equivalents of some figures shown in the manuscript.
