# Supplementary material for: Locomotion of the C60-based nanomachines on graphene surfaces
Source: Sci Rep. 2021 Jan 28;11:2576. doi: 10.1038/s41598-021-82280-7 (PMC7844297; doi:10.1038/s41598-021-82280-7)
Supplement: Supplementary file 1 — Supplementary Information. [file 41598_2021_82280_MOESM1_ESM.docx]

Supporting Information

Locomotion of the C_60_-based Nanomachines on Graphene Surfaces

S. Mahsa Mofidi,^1^ Hossein Nejat Pishkenari,*^2^ Mohammad Reza Ejtehadi,^3^ Alexey V. Akimov^4^

1. Institute for Nanoscience and Nanotechnology (INST), Sharif University of Technology, Tehran, 14588-89694, Iran

2. Mechanical Engineering Department, Sharif University of Technology, Tehran, 11155-9567, Iran

3. Department of Physics, Sharif University of Technology, Tehran, 11155-9161, Iran

4. Department of Chemistry, University at Buffalo, State University of New York 14260-3000, United States

**Table of content**

**Figure S1.** Trajectories of Nanocar and Nanotruck motion on SLG. S2

[**Figure S2.** MSD of Nanocar and Nanotruck diffusive motion. S4](#_Toc47522915)

**Figure S3.** Diffusion anomaly parameter of 4 systems vs. temperature S4

[**Figure S4.** High temperature fitting of the Arrhenius plot S5](#_Toc47522919)

[**Figure S5.** Horizontal and vertical rotation angles. S6](#_Toc47522917)

Figure S1 shows representative trajectories of Nanocar and Nanotruck molecules moving on SLG at temperatures ranging from 5 K to 1000 K. As expected, the diffusivity of both molecules increases with temperature. At 5 and 10 K, both molecules are completely motionless but at 30 K, the molecules start to show some jumps to adjacent points. If we consider the dimensions of Nanocar and Nanotruck, we realize that the motion range at 30 K is not comparable with the nanomachines' size, so it can be considered still stationary and the first signs of considerable motion appear at about 50 K.


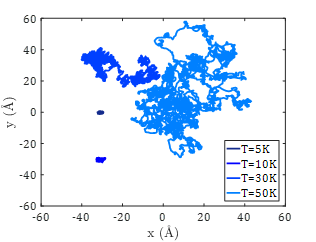

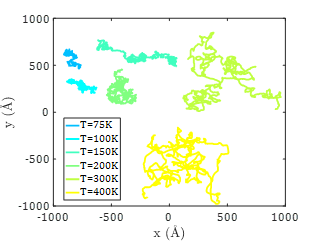

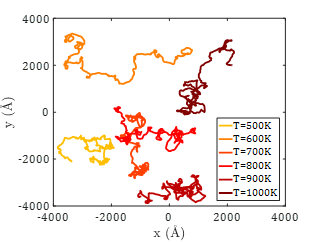


(a) (b) (c)


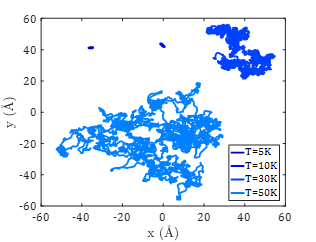

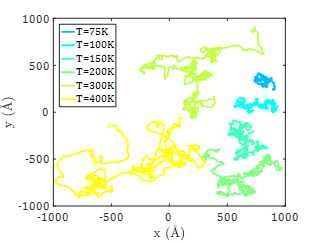

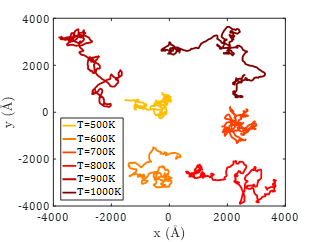


(d) (e) (f)

**Figure S1.** Representative trajectories of Nanocar (a-c) and Nanotruck (d-f) motion on SLG at different temperatures. The length of each trajectory is 40 ns.

The diffusivity is quantified by the MSD vs. time (Figure S2). To quantify the observed changes in the surface motion, we compute the diffusion coefficients, $D$, and the diffusion anomaly parameters, $\alpha$, using the long-time limit of Eq. 2 of the main text. It should be emphasized that sufficiently long trajectories must be used in the analysis. A close examination of the results suggests two distinct motion features. First, at temperatures below 50 K, molecules do not show any considerable motion, with only a few accidental jumps to adjacent sites occurring. Second, at higher temperatures, corresponding to above 300 K, the MSD exhibits a power-law dependence on time, approaching the nearly quadratic ($\alpha=1.8$ , Figure S3) limit at higher temperatures. It is due to the high kinetic energy of the molecule which overcomes the energy barrier of molecule-surface interactions easily. This type of motion can be attributed to semi-ballistic motion and Levy flights ($\alpha>1$) in Eq. 2, for which the details of surface structure (graphene hexagons) become unimportant to the dynamics.^1^ The anomaly parameter depends neither on the surface flexibility nor on the nanomachine structures (Figure S3). It is primarily determined by the temperature. The tendency for super-diffusive motion increases with temperature, with the $\alpha$ parameter approaching 1.8, at which point Levy flights appear. According to such types of motion, the molecule may travel unidirectionally over a significant distance in a relatively short time period. Such motion is semi-ballistic ($1<\alpha<2$) and is caused by a sudden deposition of kinetic energy into a translational modes.


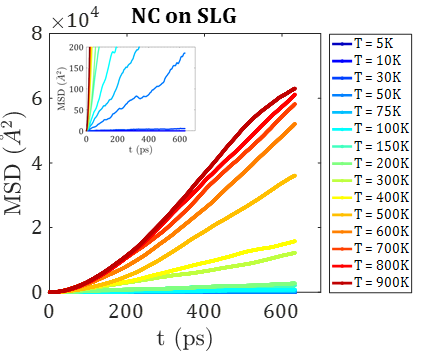

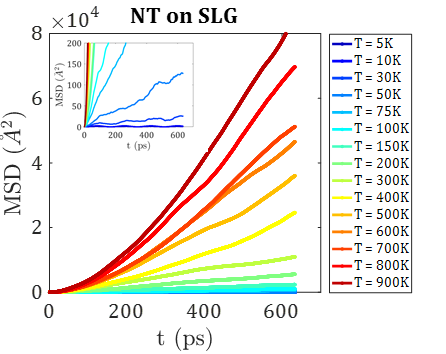


(a) (b)

**Figure S2.** The mean square displacement of Nanocar (a) and Nanotruck (b) on SLG at different temperatures. The inset zooms for the better show of low-temperature diagrams.

**Figure S3.** Diffusion anomaly parameter of 4 systems as a function of temperature

(a) (b)

(c)

**Figure S4.** High temperature fitting of the Arrhenius plot to calculate activation energy of (a) lateral diffusion, (b) pivoting motion, and (c) wheels’ rotation.

(a) (b)

**Figure S5.** Horizontal and vertical rotation angles of (a) Nanocar and (b) Nanotruck on SLG relating to a low and high temperature. At 50 K, both molecules can rotate around the vertical axis.

**References**

(1) Luedtke, W. D.; Landman, U. Slip Diffusion and Levy Flights of an Adsorbed Gold Nanocluster. *Phys. Rev. Lett.* **1999**, *82*, 3835–3838.
